# Current Visceral Leishmaniasis Research: A Research Review to Inspire Future Study

**DOI:** 10.1155/2018/9872095

**Published:** 2018-07-10

**Authors:** Kaiming Bi, Yuyang Chen, Songnian Zhao, Yan Kuang, Chih-Hang John Wu

**Affiliations:** Department of Industrial and Manufacturing Systems Engineering, Kansas State University, 2061 Rathbone Hall., Manhattan, KS 66506, USA

## Abstract

Visceral leishmaniasis (VL), one of the deadliest parasitic diseases in the world, causes more than 50,000 human deaths each year and afflicts millions of people throughout South America, East Africa, South Asia, and Mediterranean Region. In 2015 the World Health Organization classified VL as a neglected tropical disease (NTD), prompting concentrated study of the VL epidemic using mathematical and simulation models. This paper reviews literature related to prevalence and prevention control strategies. More than thirty current research works were reviewed and classified based on VL epidemic study methods, including modeling approaches, control strategies, and simulation techniques since 2013. A summarization of these technical methods, major findings, and contributions from existing works revealed that VL epidemic research efforts must improve in the areas of validating and verifying VL mathematical models with real-world epidemic data. In addition, more dynamic disease control strategies must be explored and advanced simulation techniques must be used to predict VL pandemics.

## 1. Introduction

Visceral leishmaniasis (VL), or kala-azar, is a protozoan disease that, second only to malaria in numbers of fatalities, afflicts millions of people worldwide [1]. VL is primarily distributed in East Africa, South Asia, South America, and Mediterranean Region, with an estimated 50,000 to 90,000 new VL cases each year. Ninety percent of reported VL cases occur in Brazil, Ethiopia, India, Kenya, Somalia, South Sudan, and Sudan [2]. In 2015 the World Health Organization (WHO) classified VL as a neglected tropical disease (NTD) due to relatively minimal granted attention from the public, resulting in high mortality rates (more than 20,000 in 2015), and endemic spreading in poverty-stricken regions around the world [3, 4].

VL is one of the most widespread human diseases, with more than 20* Leishmania* species identified worldwide [5]. Unlike other common forms of leishmaniasis, such as cutaneous leishmaniasis (CL), mucocutaneous leishmaniasis (ML), and post-kala-azar dermal leishmaniasis (PKDL), VL symptoms usually occur internally [1], meaning VL is more difficult to detect and cure than other leishmaniasis. Based on different kinds of susceptible species, VL can be classified as anthroponotic visceral leishmaniasis (AVL) or zoonotic visceral leishmaniasis (ZVL). AVL, which is transmitted between humans via vector carriers, is primarily caused by* L. donovani* throughout East Africa and the Middle East, especially Sudan, Somalia, Yemen, and Saudi Arabia [3]. Since most VL vaccinations for humans are still in research or minimally effective [6], AVL preventions include only early detection and treatment or use of long-term insecticide nets. ZVL, however, is transmitted between humans and other mammals, such as dogs.* L. donovani, L. infantum*, and* L. archibaldi *can result in ZVL, with specific concentrations in East Africa, South America, the Mediterranean basin, and South Asia [6]. Because dogs are the most common mammal carriers of ZVL, ZVL control strategies such as culling dogs, dog vaccinations, and use of insecticide collars are more prevalent than any AVL control strategies [7–10].

The use of mathematical models to describe and predict epidemic transmissions has become a recent trend in disease research area. Mathematical models intuitively exhibit complex VL transmission processes, and they measure variables and system parameters to reveal VL spreading dynamics and related dominating factors. Rapid advancements in computer technologies have resulted in computer-aided simulation that helps mathematical models directly predict future VL prevalence. Using results from model analysis, parametric estimation, and simulation experiments, researchers can study and anticipate disease transmission dynamics and identify disease control strategies to fight a VL pandemic. Consequently, an increasing number of studies have focused on mathematical modeling and corresponding analysis for VL disease dynamics. Approaches used in these studies can be generally categorized as system dynamic models, including ordinary differential equation (ODE) or partial differential equation (PDE) models, as well as statistic models, or machine learning models. The main contributions and results are concentrated in precise prediction tested by validation, determining the key parameters by sensitive analysis and analyzing the bifurcation point of the disease reproduction number.

A well-defined mathematical model can be used to develop disease control strategies that are ascertained by solving the mathematical model or using numerical experiments. Numerical control strategies are robust and reliable approaches because potential bias from empirical data is not included. Conversely, implementation of real-life control strategies can be cost prohibitive, irreversible, and difficult to apply in a large-scale format, especially for developing countries that lack public health resources. However, computer-aided simulations that compare possible control strategies derived from a mathematical model can be carried out, and they are relatively inexpensive and can be performed repeatedly to examine system sensitivity and determine optimal control parameters. Almost half of corresponding research used mathematical modeling approaches to study potential disease control strategies, including dog culling, use of insecticidal dog collars, vector controls, and insecticide spraying strategies. Using optimal control, parametric analysis, or stochastic control methods, research results provided well-developed guidelines for disease control centers to prevent or mitigate a VL pandemic.

**Figure 1:**
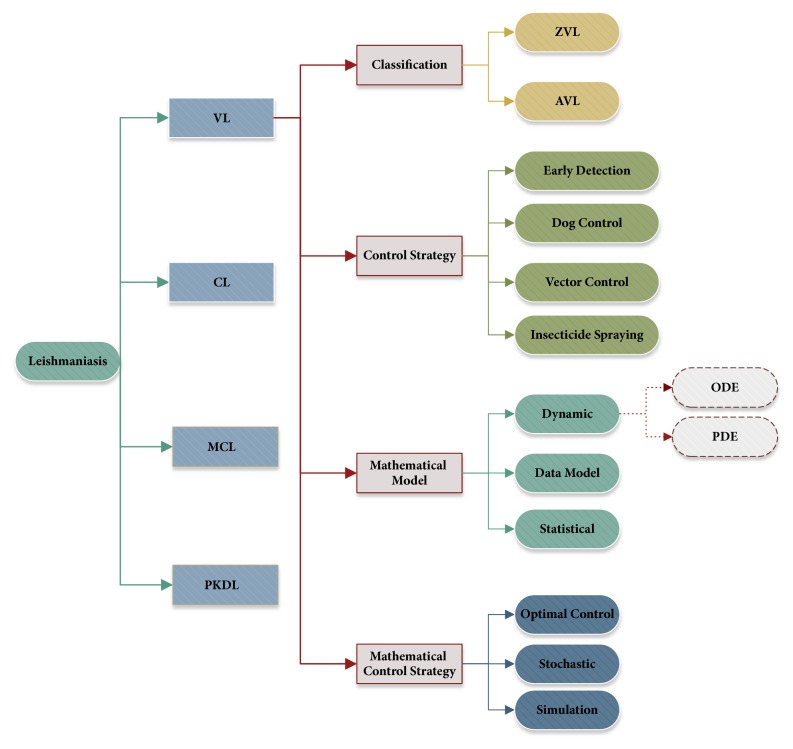
Research tree for this paper.

The rest of this paper is organized into comprehensive sections.  Section 2 introduces current worldwide VL pandemic situations, and Section 3 presents VL control strategies in existing literature and recent breakthroughs in this field.  Section 4 reviews papers on VL mathematical modeling and summarizes new developments and significant contributions since 2013.  Section 5 reviews papers on control strategies and the use of numerical simulation, and Section 6 concludes the paper and suggests possible areas for future VL pandemic research. The research tree of this paper is shown in Figure 1.

## 2. VL Pandemic Recent History

**Figure 2:**
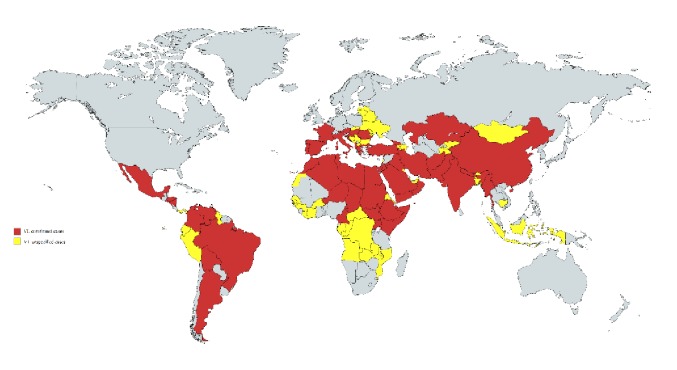
Distributions of confirmed and borderline VL cases from 1960 to 2012.

Pigott et al. collected and summarized prevalence data reports of CL and VL from 1960 to 2012 [16]. According to their summarized database, the worldwide VL pandemic has affected at least 55 countries and territories (Figure 2), and another 45 countries have reported unspecified and borderline VL cases. Affected countries are located primarily in Latin America, the Mediterranean basin, Northeastern Africa, and Asia. Moreover, VL outbreaks historically occur most often in developing or agricultural countries because citizens are more likely to be in contact with disease transmission vectors such as dogs and mosquitoes [17]. Worse still, VL traps many families in these developing countries into vicious circle. Families affected by VL have to spend more direct cost (like treatment) and indirect cost (like loss of household income) during the VL epidemic.

**Figure 3:**
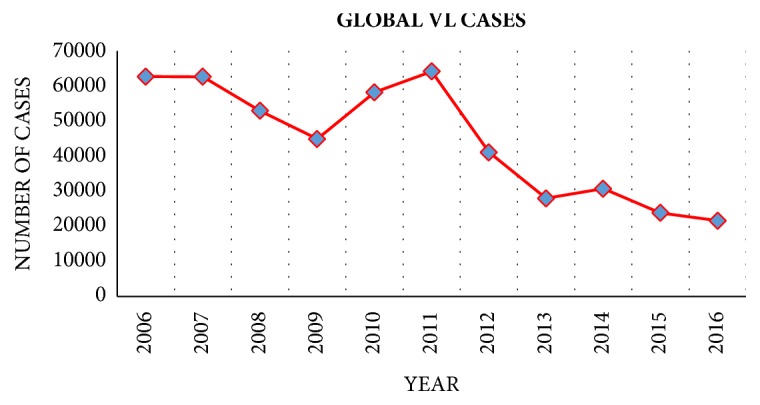
Reported VL cases from 2006 to 2016 [11].

According to the WHO neglected tropical diseases (NTD) report in 2007, VL is identified as one of the NTDs [18]. The primary reason WHO classified VL as an NTD is that confirmed VL cases have decreased worldwide from approximately 60,000 to around 20,000 since around 2010 [11], as shown in Figure 3. However, thousands of VL cases may not be covered in the WHO VL estimation report [19] since some countries without public health information systems do not submit their infection report data to WHO (e.g., Chad, the Central African Republic, and the Democratic Republic of the Congo). Even though for the countries with completed public health information system, the reported epidemiological data could be incomplete, and official figures are likely to underestimate grossly [8]. Based on the estimation, there are 500,000 new cases of VL and more than 50,000 deaths from the VL each year [17]. In 2012, another research group from the WHO Leishmaniasis Control Team corrected the number of VL estimation cases into 0.2-0.4 million, and the number of VL deaths into 20,000-40,000 [20]. Therefore, VL continues to be a serious and present threat to public health worldwide.

**Figure 4:**
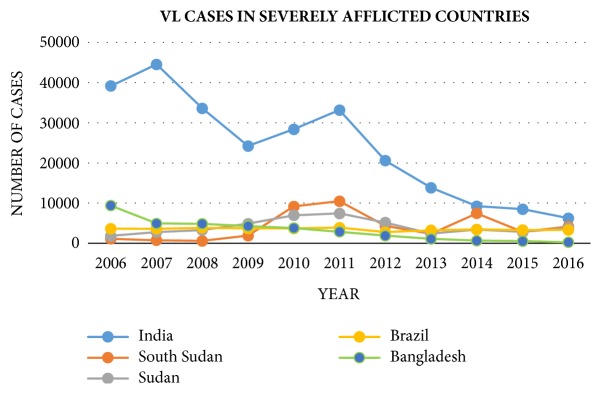
Reported VL cases in severely afflicted countries from 2006 to 2016 [11].

By observing the VL epidemiological situations for each country in Figure 4 based on the report from WHO [11], the significant drop in reported VL cases can primarily be attributed to a significant decrease in reported VL cases in India and Bangladesh. Between 2006 and 2016 the number of reported VL cases in India decreased from 39,173 to 6,249 and reported cases in Bangladesh declined from 9,379 to 255. The major reason of this decreasing is the wide utilization of insecticide-treated nets [43, 44]. However, VL prevalence did not change significantly for other countries. For example, the number of annually reported VL cases in Brazil were approximately 2,700 throughout the 10-year reporting period.

**Figure 5:**
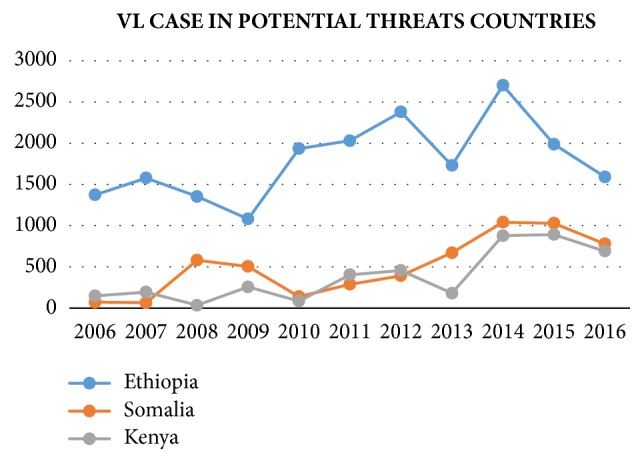
Reported VL cases in vulnerable countries from 2006 to 2016 [11].

Conversely, reported VL cases in several African countries have shown significant increase since 2006, as shown in Figure 5. For example, Somalia reported less than 100 cases in 2006 and 781 cases in 2016. Although the population of Somalia (14.32 million) is much less than the population of India (13.24 billion), the proportions of VL cases reported in these two countries were similar in 2016. If no immediate measures are taken in these selected African countries, large-scale VL outbreaks are imminent.

Therefore, VL is still a serious disease which threatens people lives and health especially in the developing countries. To strain the transmission of VL, WHO and health organizations in VL afflicted countries should apply effective prevention and control strategies. In the next section, this paper will review several existing strategies against VL.

## 3. VL Mitigation and Prevention Methods

Since 1995, researchers have focused on ZVL when investigating the intervention and prevention of VL transmission because many ZVL control strategies are related to animals. Tesh categorized former ZVL control strategies into three main classes: early detection of human cases, destruction or treatment of infected dogs, and vector control [10]. Tesh's paper mentioned that treating infected people did not affect the parasite transmission and the drug resistance of* L. infantum* made the treatment even more difficult. For the dog control, many serologically positive dogs are hard-detectible. And the VL infected dogs need expensive and continual treatments. Tesh's paper also proposed that preventing the disease in dogs population is the best control strategies, since it can interrupt the transmission cycle of ZVL. In 2002, Guerin et al. asserted that principal interventions of VL via early diagnosis and treatments, dog controls, and vector controls are effective control strategies [8]. However, Guerin et al. also pointed out that vector controls such as residual-insecticide house spraying are cost prohibitive and rarely used. Especially in India,* Phlebotomus argentipes* (the dominated species of sand fly) is becoming resistant to the insecticide. The authors also mentioned some special challenges in VL endemic areas: lack of financial support (India and Bangladesh), remote places (Brail), and civil war (Sudan). In 2006, Dantas-Torres et al. introduced the Brazilian Leishmaniasis Control Program (BLCP), which includes diagnosis and early treatment of human cases, immunological screening of seropositive dogs, and insecticide spraying [7]. Dantas-Torres et al. indicated that the culling of seropositive dogs has limited effects, as proven by experiments and mathematical models. Authors summarized several key points of unsuccessful culling dogs: the limitation of the immunological screenings to detect anti-*Leishmania* antibodies, the opposition of dog owners to the culling of asymptomatic dogs, and the lack of evidence that it is an effective intervention strategy. A paper by Quinnell et al. suggested that tropical insecticides-collars and pour-ons can be used to reduce VL incidences for dogs by more than 83% [9]. However, like the insecticide spraying, the cost of this strategy limits the feasibility of the tropical insecticides-collars. The authors offered an alternative method, insecticidal bath, which can protect dogs for at least 3.5 months against* Phlebotomus chinensis*.

In 2014, Werneck considered the effectiveness of control strategies on the basic reproduction number *R*_0_ [22]. The author found that vector controls (e.g., controlling vector density, the ratio of female vector, and the extrinsic incubation period of* L. infantum* in sand flies) and dog controls (e.g., culling infected dogs, dog vaccinations, and insecticide-releasing dog collars) can cause the disease reproduction rate *R*_0_ to decrease to less than 1, meaning that the number of infected individuals will eventually decrease to zero. However, the author did not find the relevant data to support these control strategies. He pointed out some potential implementation difficulties for these strategies, such as costs issues associated with the continual uses of tropical insecticides-collars. Werneck also voiced concern that the effectiveness of using insecticide collars in the large community (like Brazil) may not work so well, since the insecticide collars strategy has the relatively short-term effect and consequent need.

Due the high cost of indoor residual spraying, insecticide-treated nets (ITNs) were introduced as an alternative control strategy for ZVL [21]. Experimental trials in sub-Saharan Africa, Latin American, Thailand, Pakistan, and Iran show that ITNs could reduce infections with malaria by 17%–62%. In 2015, Picado et al. summarized the results of the KALANET project to analyze ITN effectiveness. The KALANET project is a cluster-randomized controlled trial in India and Nepal [23]. The KALANET project was conducted in 26 high-incidence regions (in India and Nepal) with more than 20,000 inhabitants observed over 24 months. Results showed that use of long-lasting insecticidal nets resulted in a 50% reduction of* L. donovani* infections. However, this study also showed that some of those nets were untreated, many were damaged, and most families did not have enough nets to protect all family members. The authors suggested standardizing the color and size of insecticide nets; they also want the government to replace the untreated and damaged nets by new treated nets for free for publics. Although the long-lasting insecticidal nets can prevent VL transmissions while people are sleeping, recent entomological findings in India indicated that vectors are more exophilic than previously thought, meaning that people engaged in outdoor work have more opportunities to become infected [45].

In 2016, Özbel et al. analyzed the geographical distribution, ecological aspects, and species habits of VL vectors (sand flies) in Bangladesh [24]. In general, two genera of sand fly (*Phlebotomus* and* Sergentomyia*) and a total of 13 species were recorded in Bangladesh, among which* Phlebotomus argentipes *is the dominant vector species in VL endemic areas of Bangladesh (>94%). Researchers also found that the* Phlebotomus. argentipes *population peaks around monsoon season and reaches the lowest ebb during winter and summer in Bangladesh. They also determined that eight ecological parameters (soil temperature and moisture, rainfall, air temperature, relative humidity, soil pH, soil organic carbon, and wind speed) can influence the* Phlebotomus. argentipes* population. Future research must enact control strategies based on their ecological aspect. The potential application of this research can provide early warning of the incoming VL epidemic and narrow the range of the insecticide spraying.

As the most effective control strategy for infectious diseases, the successful vaccination on VL is long-awaited for the VL afflicted countries. The experiment on VL vaccination was started in 1990s; researchers tried to utilize the proteins from* L. donovani* to develop vaccines [46]. At the beginning of the 21st century, researchers investigated the possibility of vaccination based on DNA and genetically attenuated parasites [47, 48]. More recently, Duthie et al. studied several different vaccine antigens for VL transmissions using recombinant proteins from* E. coli* [25]. Their research results have shown that several potential vaccine antigen candidates are verified in different platforms. The authors consolidated seven vaccine candidates as recombinant proteins in* E. coli*, and they validated the effectiveness of* E. coli* to* L. donovani *via experiments on mice. However, their research pertained to only nonhuman experiments. Significant research and advancements are still needed to obtain effective vaccination for humans against VL parasites.

For now, even though many contributions have been done for the VL controls and prevention, an effective, feasible, and economical control strategy is still an ongoing effort. The current VL control strategies and corresponding deficiencies are summarized in Table 1. Since the most of VL severely afflicted countries are developing countries, how to balance the effectiveness and costs involved in such VL control plan is delicate tradeoff. This paper will discuss more about this particular issue in Section 5.

## 4. Mathematical Epidemiology Models for VL

### 4.1. System Dynamic Model

In 1996 Dye first introduced a 4-equation ODE susceptible-latent-infectious-removed (SLIR) model to describe the VL epidemic [49]. SLIR, respectively, represents the susceptible, latent, infectious, and resistant populations of VL, and the model considered the transitions between these populations. However, the author did not consider the behaviors of dog and vector in his model. Courtenay et al. improved Dye's model by considering the dog population as a key parameter in the SLIR model [50]. Variations of this parameter directly influence the human infection rate. Ribas et al. built an ODE model to describe VL transmissions among humans, dogs, and vectors [14]. The model utilized 11 ODE equations, including the susceptible (dog, sand fly, and human), latent (dog, sand fly, and human), infectious (dog, sand fly, and human), and recovered (dog and human) populations. Although their model was presented without data, simulation, and analysis, it was the first model to describe behaviors for all species involved in VL.

**Figure 6:**
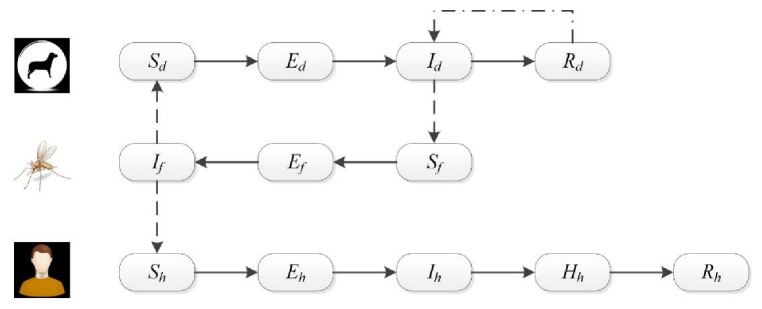
System diagram of ZVL transmission model [12], where *d*, *f*, *h* represent the dog, sandflies, and human species and *S*, *E*, *I*, *R*, *H* represent the susceptive, exposed, infected, recovered, and hospitalized population for each species.

Since WHO's designation of VL as an NTD in 2015 [51], an abundance of research and studies have focused on developing mathematical models of VL. In 2016 Zhao et al. developed a 12-equation ODE model to comprehensively describe a VL epidemic (Figure 6) [12]. They improved the model by including a hospitalized population into the ODE system; this population has a higher probability of survival than infections without hospitalization due to the systematic treatment. The authors utilized a backward bifurcation method to analyze VL equilibrium behavior and the basic reproduction rate *R*_0_. Their research showed that VL equilibrium is highly related to a critical model parameter, *R*_*c*_, as the epidemic threshold value for *R*_0_. Similarly, Subramanian et al. proposed a compartment-based ODE model of VL transmission to explain disease transmissions in symptomatic VL, asymptomatic VL, and PKDL-infection classes [26]. Sensitivity analysis of model parameters found that the biting and birthing rates of sandflies and the recovery rate of symptomatic humans are dominating factors for VL epidemic control.

Biswas simplified the 12-equation ODE model from Zhao et al. to an 8-equation ODE model by dividing the nonhuman populations (dog and vector) into susceptible and infected population groups [27]. With a simplified ODE model, researchers reduced the complexity of system sensitivity analysis and reduced the numbers of assumed or estimated model parameters. This model also successfully reproduced the 2011 VL epidemic in South Sudan. Shimozako et al. transferred the ODE model in Ribas et al. by considering the dog population as the only source of infection since vectors could not transfer ZVL without dogs [28]. Therefore, their mathematical model contained eight variables corresponding to the susceptible, latent, infectious, and recovery populations for dogs and humans. Le Rutte et al. compared three ODE models and corresponding simulation results while considering indoor residual spraying [29]. The primary difference between the three models was how relationships between PKDL and the recovery population were modeled. Their research predicted that, using 60%–80% IRS coverage, VL epidemics could be eliminated within three years in Bihar, India. In addition, other researchers have made incremental contributions using various ODE VL epidemic models [30–33].

Although many ODE models describe VL epidemics and transmission, the development of a novel dynamic model is an active area of research in the investigation of complicated transmission behaviors of VL under various situations and the development of improved mitigation and control strategies. Bi et al. introduced a two-dimensional PDE model based on an existing ODE model [13]. The model presented in their research considered human age structures and time as two dimensions since historical data have strongly suggested that VL infection rates are highly correlated to human age groups. For example, children and teenagers (aged from 0 to 20) are more likely to become infected compared to other age groups. This research used computer simulation and mathematical analysis to explain this phenomenon and recover the historical VL endemic data published by WHO.

### 4.2. Models Based on Real-World Data

VL attracted significant epidemiology research attention; abundant statistical data were collected and reported on the current VL pandemic worldwide by scholars and researchers. Many researchers realized the importance of data utilization in VL model development. Disease data is generally utilized in three ways: use of reported data to build statistical models, use of historical data to predict future prevalence, and use of existing data to calibrate model parameters in mathematical epidemic models.

The primary objective of building a VL statistical model is to statistically identify key parameters in the VL transmission process and determine relationships between the number of parameters and the number of infected population. Werneck et al. used consolidated census tracts to analyze VL disease prevalence data from different regions of Brazil [52]. The authors developed a spherical covariance structure model based on census data from 1992 to 1996 in Teresina, Brazil. By exploring spatial correlation structures of the census data, they found a positive correlation between reported VL incidences and residence in areas of green vegetation, especially in favela. In 2007 Werneck* et al.* extended their previous work by analyzing and comparing the results from 21 statistical models [53]. According to human and canine VL data in Teresina, Brazil, the study found significant correlations between residence in areas with green vegetation and infected dogs and between the human infection rate and urbanization index or socioeconomic status index.

Thompson et al. studied relationships between climate and VL epidemics by establishing the statistical regression model [54]. Their research found that rainfall is the most significant parameter statistically correlated to VL incidences. The influence of geographical features of areas of residence (e.g., cities, plains, or foothills) on VL transmission was also considered. The foothill population statistically revealed a higher risk of VL infection than other populations. De Araújo et al. considered the statistical model with data and found that spatial data is more reliable and accurate for VL epidemic study and analysis [34]. Therefore, they applied spatial statistical modeling and the Bayesian approach to model and estimate risks of VL using historical data from Belo Horizonte, Brail. Their research showed that the relative risk of VL is correlated to income, education, and the ratio of infected dogs to inhabitants in Belo Horizonte. However, as opposed to their previous research findings, residence in areas with green vegetation did not show significant correlations to the risk of VL.

Ecological niche modeling (ENM), stemming from the genetic algorithm [55], has been widely used to predict VL prevalence since 2006. Nieto et al. first used ENM to analyze VL data from northeastern Brazil [56]. Using the geographic information system (GIS), the ENM model can predict prevalence risks in three levels (high, moderate, and low). When being validated with historical data from Bahia, Brazil, the predictive model demonstrated high accuracy (more than 90%) on high-level and moderate-level data. Similar approaches were used to predict VL prevalence in North America and Middle Eastern regions [57, 58]. Several other methods were utilized to predict the trend of VL epidemics with various factors. Elnaiem et al. summarized data from eastern Sudan in GIS and then used that data to build predictive models of VL infections based on rainfall and corresponding distance to a river [59]. Oshaghi et al. built a predictive degree-day model for VL using the single triangulation method [60]. This model predicted temporal and spatial distribution of VL infection density and generations of sandflies. Karagiannis-Voules et al. employed Bayesian geostatistical models to fit the VL incidence data from Brazil, and they identified environmental and socioeconomic predictors using Bayesian variables [15]. Their research results predicted that regions with humid climates and dense vegetation distributions are more vulnerable to VL than other regions.

**Figure 7:**
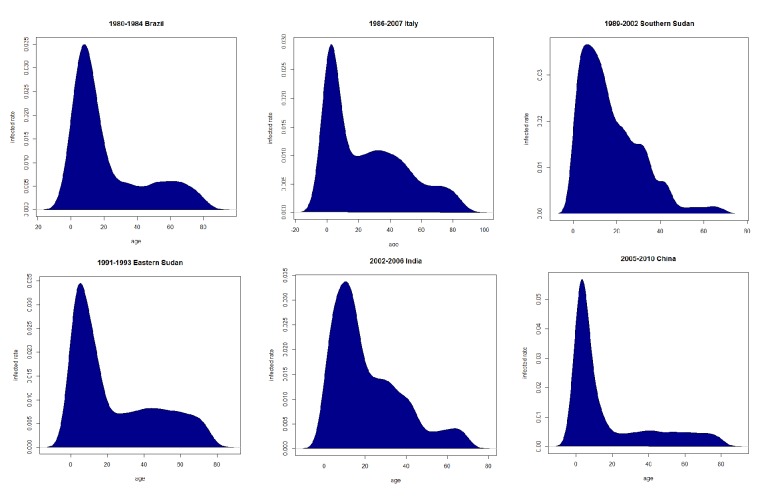
Infection rate distribution based on human age in various countries [13].

Parameter estimation is another essential application when validating VL mathematical models using real-world data since the use of assumed system parameters in the model may reduce model reliability. Bi et al. summarized age structures of VL infections in various regions [13]. They revised the constant infection rate into age-dependent distribution of infection rates by studying historical VL prevalence data from existing literature from diverse regions over time (Figure 7). Use of this age-dependent distribution function allowed their PDE epidemiology model to reflect VL human prevalence in both age and time periods. Biswas et al. established the posterior distribution of different parameters and initial parameters based on observation data [27]. Use of parameter distribution allowed the simulation experiments to reflect more than one result with unique possibilities. Even though most mathematical models still use assumptions or estimations with existing literature as their system parameters [12, 26, 28, 29], an increasing number of studies are utilizing real-world data to more accurately estimate system parameters and validate their models.

## 5. Optimal Control Strategies Using VL Models

### 5.1. Parameter Control Strategy

The most generalized control strategy in a VL mathematical model is the parameter control strategy, which assumes that the key parameters in the model are adjustable. When the parameters are adjusted, the model outputs become dependent variables; therefore, the parameter adjustment process can be considered a corresponding real-world control strategy. In 2002 Courtenay et al. introduced the numerical control strategy to the field of VL mathematical modeling [50]. They assumed that the parameter of dog density could be controlled by culling dogs in specific areas, and their research findings showed that dog culling could effectively reduce the proportion of infectious population. Ribas et al. improved the parameter control method by considering additional control parameters [14], including the dog treatment rate, the insecticide collar utilization rate, the dog culling rate (natural mortality rate of dogs), the dog vaccination rate, and the mortality rate of sandflies (vector control). Using the control parameter method, they used simulation experiments to compare the efficiencies of each control strategy, as shown in Figure 9, where vector control proved to be more effective than control strategies such as culling and vaccine. Shimozako et al. incorporated control strategies such as dog treatment, dog vaccination, and use of insecticidal dog collars into an ODE mathematical model of VL [37]. However, they increased the dimension of the model by using control variables to replace the control parameters in their model. A cost-effect analysis and simulation experiments showed that use of insecticidal dog collars should be the most utilized control strategy.

### 5.2. Optimal Control Strategy

Lev Pontryagin established the optimal control strategy in the 1950s [61, 62]. This strategy provides optimality criterion by maximizing or minimizing a given objective function subject to constraints defined in the differential equation mathematical model. Zhao et al. introduced optimal control into their 12-equation ODE mathematical model of VL [12], including the susceptible, latent, and infectious populations for sandflies, humans, and dogs; recovered human and dog populations; and hospitalized human population. Their study included three control strategies in the model: the control dog prevention level (vaccine protection or dog culling), the control insecticide usage level (insecticide sprayed around buildings), and the control personal protection level (long-lasting insecticide). Consideration of exposed human, infected human, and sandfly populations as well as control cost as the control objective function caused the final control strategy to effectively lower the negative influence of VL by approximately 80% (Figure 9). Agusto et al. considered the use of fabrics and insecticidal animal collars as an additional control [33]. Their research was innovative because they used various combinations of controls instead of utilizing control strategies identical to previous research. Their simulated experimental results showed that use of an ODE mathematical model allowed their control strategies to effectively reduce VL and PKDL infected human populations. Biswas et al. revised a previous optimal control by suggesting a strategies mechanism and incorporating various combinations of optimal strategies into the ODE mathematical model with a given objective function [36]. This objective function was a linear combination of VL and PKDL infections with the cost of control strategies. They calculated the corresponding infection averted ratios (IAR) and incremental cost-effectiveness ratios (ICER) for each control strategy, where IAR is the ratio of the number of infections averted to the number of recoveries and ICER is the additional cost per additional health outcome. The researchers then selected the strategy with the highest IAR and lowest ICER as the optimal strategy. An optimal control strategy specifically targeting the human VL vaccination was also analyzed, but no evidence revealed the effectiveness of a human VL vaccination [27].

### 5.3. Control Strategy Selection Using Simulation

**Figure 8:**
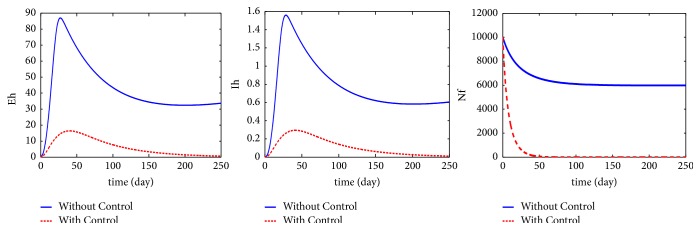
Simulation of dog culling [12].

Simulation comparison is the most common method of VL mathematic control modeling in simulation.  Figure 8 compares the human infected population with control (dog culling, sandfly control, and human protection) and without control [12]. The solid line in the figure shows epidemic performance (which includes the exposed human *E*_*h*_, the infected human *I*_*h*_, and the total population of sand flies *N*_*f*_) of the control strategy, which reduced disease prevalence to 80%. This simulation proved the effectiveness of the combined control strategies.

**Figure 9:**
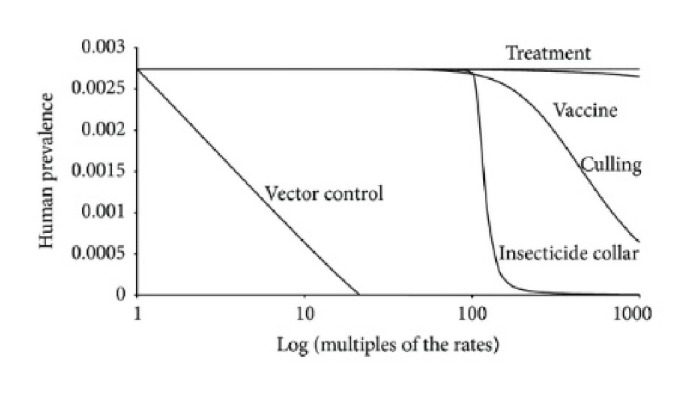
Efficacy comparison of control strategies [14].

Efficacy comparison between control strategies is another type of simulation comparison. Ribas et al. compared the human prevalence influence using vector control, insecticidal collars, dog culling, dog vaccines, and dog treatment [14], as shown in Figure 9. When the parameter was changed to manipulate control levels, the human prevalence by vector control decreased most rapidly, proving that human prevalence is the most sensitive to vector controls. Therefore, vector control is the most effective control strategy.

**Figure 10:**
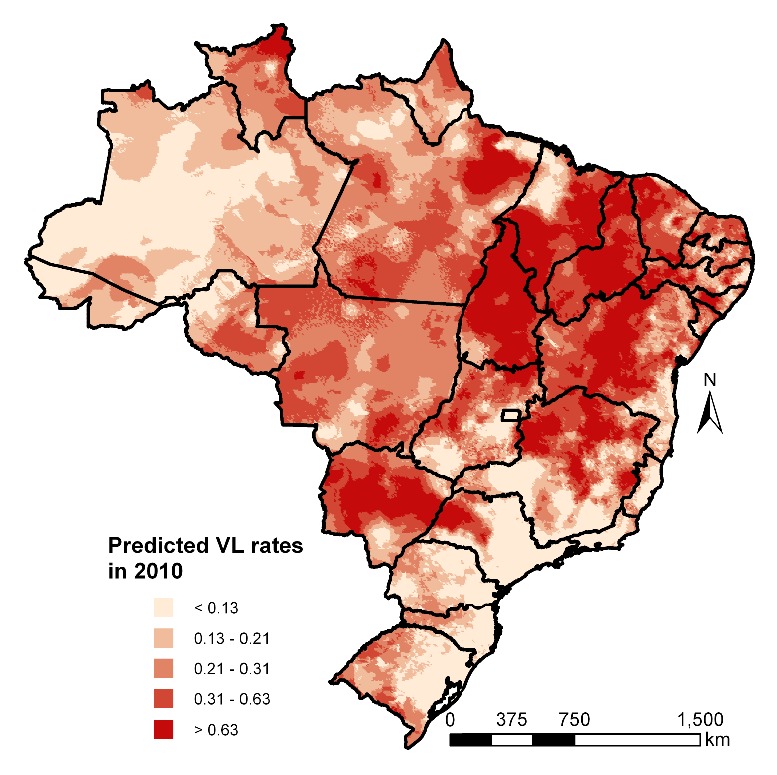
Spatial simulation of predicted VL rates in 2010, Brazil [15].

Spatial simulation is a simulation estimation method that provides spatial information throughout the model behaviors. Using GIS, spatial simulation can exhibit VL prevalence information from various regions, as shown in Figure 10. Karagiannis-Voules et al. utilized historical data (2001–2009) from Brazil to build a statistical model [15]. Their simulation used GIS to predict VL prevalence in Brazil in 2010, thereby directly reflecting high infection density in the eastern region of Brazil.

## 6. Discussion and Conclusion

Although globally reported, VL-confirmed cases have decreased since 2011; VL prevalence has not improved significantly worldwide except in South Asia (i.e., India and Bangladesh). However, VL outbreaks have increased in Ethiopia, Somalia, and Kenya since around 2008. Moreover, public health agencies in underdeveloped African countries such as Chad and the Central African Republic do not have resources and capabilities to collect and report VL incidences. However, because these countries are near regions severely afflicted with VL, such as Sudan and South Sudan, the number of global VL cases reported from WHO may be underestimated.

This paper reviewed current VL epidemiological research ranging from VL epidemic control strategies to VL mathematical models and related optimal control strategies. The research demonstrated how to use numerical methods such as modeling and sensitivity analysis, as well as equilibrium/stability studies and simulation experiments, to assist mitigation and prevention strategies for a worldwide VL pandemic. Governments and health organizations can utilize the modeling and simulation results to predict or estimate impacts of various control strategies.

Despite significant research efforts using mathematical models for the VL epidemic, research gaps still exist and many areas of study remain unexplored.  Table 2 summarizes 21 literature works related to mathematical modeling and VL disease control strategies since 2013. Most of the reviewed research used or proposed system dynamic mathematical models or statistical models; approximately half of these research works considered real-world data and studied possible control strategies. Only one paper included real-world data, control strategies, mathematical models, and numerical simulation experiments.

Future work, thorough VL epidemic research using mathematical or statistical models, ought to consider the four following aspects:

1. building more sophisticated mathematical models to explain underlying infectious disease transmission dynamics,
2. including real-world data to aid model validation and verifications,
3. exploring possible disease control/mitigation strategies to increase understanding of model maneuverability and robustness,
4. using numerical simulation experiments as a predictive tool to verify the feasibility of model and control strategies.

Moreover, future work in these four aspects of VL mathematical modeling must utilize modern analytical tools. The disadvantage of current modeling is the limited diversity of model types. A majority of existing VL mathematical models are ODE models, which are widely used but produce limited predicted results without details. Therefore, more statistical, machine learning, and PDE models are needed to build sophisticated, comprehensive mathematical models of VL. Statistical and machine learning models can more advantageously utilize real-world data to ensure model prediction accuracy, while use of a PDE model can enrich predicted results with age, gender, socioeconomic group, ethics, and spatial information. For the second aspect, the inclusion of real-world data, most test data currently used to validate and verify underlying mathematical models are estimated or assumed, consequently limiting the mathematical model to reflect only data from previous VL epidemic episodes. Future research efforts should utilize recent epidemic data with temporal and spatial data during the modeling phase, making the modeling process increasingly dynamic and reflecting real-time data while predicting possible trends of an ongoing epidemic. The current primary disadvantage of the third aspect, exploring possible control strategies, is that the control strategies lack of applicability in the real world. In fact, the most effective control strategies suggested by the mathematical models may not be operable or they may be too cost prohibitive to be implemented. Operable control strategies should be carefully quantized, such as specific consideration of the optimal level of canine culling in a particular time frame or the level of insecticide spraying in each area affected by VL. For the fourth aspect, current studies using numerical simulation experiments frequently provide insufficient information from simulation results. Most simulations of VL models can only predict the trend of VL infections. Future research should focus on spatial simulation and agent-based simulation as well as the study of the interactions between multiple regions or environments.

In conclusion, the use of mathematical models to study, analyze, and predict VL epidemics and to explore effective and implementable control strategies remains an active and study-worthy area of future research. However, research results from more comprehensive studies that use modern analytical tools will help public health organizations understand and prevent the VL disease.

## Figures and Tables

**Table 1:** Current VL control strategies and corresponding deficiencies.

Strategy	Category	Deficiency	Reference
Early detection	Human control	Doesn't affect the parasite transmission	[10]
Culling dogs	Dog control	Opposition of dog owners & Hard to detect infected dogs	[7, 10]
Dog treatments	Dog control	Expensive	[9]
Canine vaccination	Dog control	Expensive & Drug resistant	[9]
Spraying insecticide	Vector control	Expensive & Drug resistant	[8, 21]
Immunological screening of seropositive dogs	Dog control	Expensive & Needs high level technique support	[9, 10]
Insecticide collars	Dog control	Expensive & May not work in large community	[22]
Insecticide-treated nets	Vector control	Damaged and untreated nets have low effectiveness	[23]
Ecological control	Vector control	Needs more time to be applied in the real world	[24]
Vaccination control	Human control	Not available at the current time	[25]

**Table 2:** Recent papers on mathematical modeling of VL.

Paper	Published Year	Real Data Involved	Control Strategies	Transmission Models	Simulation
Ribas et al. [14]	2013	No	Yes	Yes	No
Zhao et al. [12]	2016	No	Yes	Yes	Yes
Subramanianet al. [26]	2015	Testing model	No	Yes	Yes
Biswas et al. [27]	2017	Parameter estimated	Yes	Yes	Yes
Shimozako et al. [28]	2017	Testing model	No	Yes	Yes
Le Rutte et al. [29]	2017	Testing model	No	Yes	Yes
Costaet al. [30]	2013	No	No	Yes	No
Sevá et al. [31]	2016	No	No	Yes	No
Zouet al. [32]	2017	No	No	Yes	Yes
Agustoet al. [33]	2017	No	Yes	Yes	Yes
Bi et al. [13]	Not yet	Parameter estimated	No	Yes	Yes
de Araújo et al. [34]	2013	Yes	No	No	No
Karagiannis-Voules et al. [15]	2013	Yes	No	No	Yes
Miller et al. [35]	2014	Yes	Yes	Yes	No
Biswas et al. [36]	2017	Testing model	Yes	Yes	Yes
Shimozako et al. [37]	2017	Testing model	Yes	Yes	Yes
Stauch et al. [38]	2014	Testing model	No	Yes	Yes
Zamir et al. [39]	2017	No	No	Yes	Yes
Boukhalfaet al. [40]	2017	No	No	Yes	Yes
Gorahavaet al. [41]	2015	No	Yes	Yes	No
Rock et al. [42]	2016	No	Yes	Yes	Yes
